# The role of the E3 ligase Not4 in cotranslational quality control

**DOI:** 10.3389/fgene.2014.00141

**Published:** 2014-05-19

**Authors:** Olesya O. Panasenko

**Affiliations:** Department of Microbiology and Molecular Medicine, Institute of Genetics and Genomics of Geneva – University Medical Center, Faculty of Medicine, University of GenevaGeneva, Switzerland

**Keywords:** protein quality control, ubiquitination, proteasome, translation, RNA decay, ubiquitin proteasome system, protein degradation, E3 ligase

## Abstract

Cotranslational quality control (QC) is the mechanism by which the cell checks the integrity of newly synthesized proteins and mRNAs. In the event of mistakes these molecules are degraded. The Ccr4-Not complex has been proposed to play a role in this process. It contains both deadenylation and ubiquitination activities, thus it may target both aberrant proteins and mRNAs. Deadenylation is the first step in mRNA degradation. In yeast it is performed by the Ccr4 subunit of the Ccr4-Not complex. Another complex subunit, namely Not4, is a RING E3 ligase and it provides the ubiquitination activity of the complex. It was found associated with translating ribosomes. Thus, it has been suggested that Not4 is involved in ribosome-associated ubiquitination and degradation of aberrant peptides. However, several other E3 ligases have been associated with peptide ubiquitination on the ribosome and the relevance of Not4 in this process remains unclear. In this review we summarize the recent data and suggest a role for Not4 in cotranslational protein QC.

## QUALITY CONTROL

The cell proteome is highly dynamic and has to be controlled both qualitatively and quantitatively to maintain the survival of the organism. It can be monitored at multiple levels, including gene expression, mRNA metabolism, protein production, and, finally, protein degradation. In the cytoplasm, ribosomes bind to mRNAs that carry the genetic information and use them as a template for determining the correct sequence of amino acids in a particular protein. Errors regularly occur during translation resulting in defective translational complexes, ribosome stalling (delaying) on the mRNA, and the production of defective non-functional proteins. To prevent the accumulation of such aberrant products cells have developed cotranslational quality control (QC) mechanisms that promote disassembly of stalled ribosomes and recognize and degrade defective mRNAs and proteins [reviewed in (Parker, 2012; Shoemaker and Green, 2012; Lykke-Andersen and Bennett, 2014)]. Ribosome stalling occurs as the result of at least three mRNA QC pathways. Stable mRNA hairpin structures, rare codons and positively charged polylysine or polyarginine tracts activate the no-go decay (NGD) pathway. The absence of a termination stop codon causes non-stop decay (NSD). Premature translation termination activates nonsense-mediated decay (NMD). Defective mRNA fragments are first deadenylated at the 3′ and decapped at the 5′ end of the molecule, before being degraded by the corresponding exonucleases. Defective polypeptides produced as a result of ribosome stalling are recognized and degraded by the ubiquitin–proteasome system (UPS; Ciechanover, 1998; Goldberg, 2003; Finley et al., 2012). The UPS involves the proteasome, a highly conserved large multicatalytic protease that degrades misfolded proteins or proteins whose presence in the cell is no longer needed. The proteasome recognizes an ubiquitin signal on the target protein whose attachment requires an ubiquitin-activating enzyme, E1, an ubiquitin-conjugating enzyme, E2, and an ubiquitin-protein ligase, E3. Dysfunction of the UPS causes the accumulation of non-functional misfolded proteins, which can aggregate, be toxic for the cell, and, in extreme cases, lead to the cell death.

**FIGURE 1:**
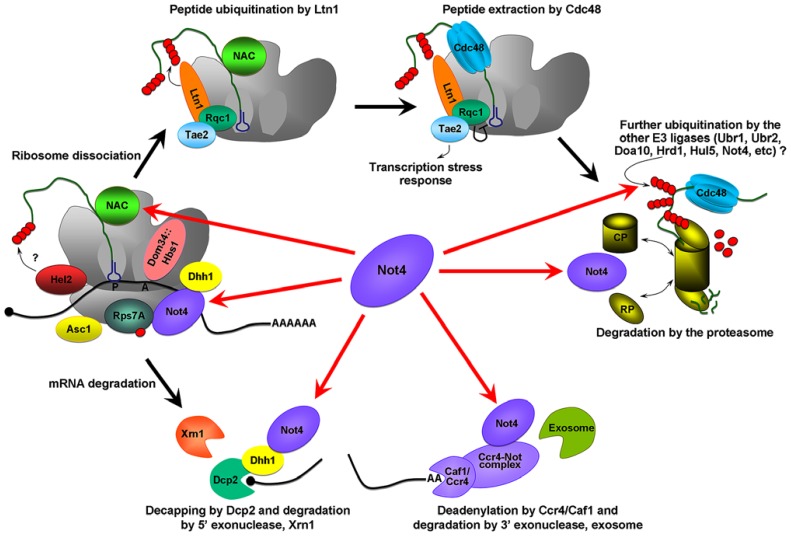
**Possible roles of Not4 in cotranslational QC**. The Hbs1/Dom34 complex binds the empty A site on the stalling ribosome and stimulates the endonuceolytic mRNA cleavage, and separation of the stalled ribosomes. The 40S ribosome binding protein Asc1/RACK1, and E3 ligase, Hel2, were identified as proteins involved in QC and acting before ribosome dissociation (Kuroha et al., 2010; Brandman et al., 2012; Duttler et al., 2013). Hel2 could initiate ubiquitination of the nascent chain. Further ubiquitination is associated with Ltn1 (Defenouillere et al., 2013). Ltn1 together with proteins Tae2 (translation-associated element 2), Rqc1 (ribosome quality control 1), and the disaggregase Cdc48 forms a RQC complex (ribosome QC complex; Brandman et al., 2012; Defenouillere et al., 2013). Cdc48 extracts the peptide from the 60S, Rqc1 regulates the activity of the RQC by a negative feedback loop, and Tae2 signals translational stress to the corresponding transcription factors (Brandman et al., 2012). Extracted peptides are probably subjected to further ubiquitination by other E3 ligases and finally degraded by the proteasome. The red arrows indicate the possible pathways by which Not4 may act. Not4 may ubiquitinate translationally arrested peptides. It may also be involved in the clearance of arrested protein products by the proteasome. Not4 may preserve translation acting through several targets; the deadenylase module of the Ccr4-Not complex, Dhh1, the ribosome-associated chaperone, NAC, and through the ubiquitination of Rps7A. Finally, Not4 may interact with mRNA and “sense” ribosome pausing and/or recruit the factors necessary for correct translation.

When ribosomes stall the Hbs1/Dom34 complex binds the empty A site and stimulates both mRNA cleavage, and separation of the stalled ribosomes [reviewed in (Lykke-Andersen and Bennett, 2014) and (**Figure 1**)]. Some E3 ligases, such as Hel2, are associated with the 40S ribosome (Kuroha et al., 2010; Brandman et al., 2012; Duttler et al., 2013) and could initiate ubiquitination of the nascent chains. Further ubiquitination of the polypeptide occurs via the E3 ligase Ltn1 (Defenouillere et al., 2013). Ltn1 together with several other proteins forms the RQC complex (ribosome QC complex; Brandman et al., 2012; Defenouillere et al., 2013), that extracts the peptide from the 60S ribosome. Extracted peptides are probably subjected to further ubiquitination by other E3 ligases and finally degraded by the proteasome.

Up to 50% of newly synthesized proteins may be ubiquitinated and degraded cotranslationally (Schubert et al., 2000; Turner and Varshavsky, 2000; Qian et al., 2006). Even taking into account later studies (Duttler et al., 2013; Wang et al., 2013) reporting that these numbers may be overestimated, ribosome-associated degradation is nonetheless very abundant and crucial. However, which E3 ligase(s) is responsible for the ribosome-associated ubiquitination remains an open question. Several candidates have been proposed for this role. One of these is Not4, a subunit of the Ccr4-Not complex (Dimitrova et al., 2009). However, later studies also questioned its role in cotranslational QC.

## THE Not4 E3 LIGASE OF THE Ccr4-Not COMPLEX

Not4 is an E3 ligase of the RING family type that catalyzes protein ubiquitination. In yeast Not4 in association with eight other subunits forms the Ccr4-Not complex [reviewed in (Collart and Panasenko, 2012; Collart, 2013)]. This complex is involved both in nuclear transcriptional regulation and mRNA deadenylation and decay [reviewed in (Collart, 2003; Collart and Timmers, 2004; Miller and Reese, 2012)]. At the N-terminus Not4 carries a Zn-binding RING domain that is important for ubiquitination activity. The region responsible for its interaction with the rest of the Ccr4-Not complex is located between amino acids 430 and 450 (Panasenko and Collart, 2011). Two other domains, a coil-coiled domain and an RNA recognition motive (RRM) have been predicted but there functions are as yet unknown.

For ubiquitination, Not4 cooperates with the Ubc4 and/or Ubc5 E2 enzymes. Amongst several identified Not4 substrates (Collart, 2013) there are proteins linked to the ribosome and translation. These are the small ribosomal protein, Rps7A (Panasenko and Collart, 2012) and a ribosome-associated chaperone, the nascent polypeptide-associated complex, NAC (Panasenko et al., 2006, 2009). Ubiquitination of these substrates does not lead to degradation but may instead play an important role in their function. For example, ubiquitinated Rps7A was present only in 80S monosomes and in polysomes and its ubiquitination was important for cell viability (Panasenko and Collart, 2012). The ubiquitinated site on NACβ is located on the ribosome-binding loop of the protein. Thus, it has been suggested that the Not4 mediated ubiquitination contributes to NAC-ribosome association (Panasenko et al., 2009).

Recent studies have revealed that Not4 is involved in the assembly of the proteasome (Panasenko and Collart, 2011). The proteasome is composed of two main subcomplexes, the core particle (CP), and the regulatory particle (RP) (Finley et al., 2012). RP recognizes the substrates, while CP performs the cleavage. Appropriate RP–CP interaction is essential for normal proteasome function. Proteasome assembly is not yet fully understood. However, it has been established that RP and CP assembly requires several chaperones (Bedford et al., 2010; Kish-Trier and Hill, 2013). It was shown that Not4 interacts with RP subunits and proteasome chaperones and is important for normal proteasome activity and integrity (Panasenko and Collart, 2011).

## Not4 IS INVOLVED IN COTRANSLATIONAL QC

The presence of Not4 on the polysomes (Dimitrova et al., 2009; Panasenko and Collart, 2012) and its interaction with ribosomal and ribosome-associated proteins led to the idea that it may be involved in cotranslational QC and NGD. The role of Not4 in NGD was suggested because arrested proteins encoded by mRNAs containing rare codons or polybasic tracts accumulated in *not4Δ* mutants (Matsuda et al., 2014). It was proposed that Not4 may ubiquitinate the aborted proteins appearing during translational arrest and that this would lead to their degradation by the proteasome (Dimitrova et al., 2009). However, subsequent studies demonstrated that the ubiquitination of these aberrant products occurred via the E3 ligase Ltn1, the yeast homolog of Listerin, a protein involved in neurodegeneration in mice (Bengtson and Joazeiro, 2010). This E3 ligase interacts with the 60S ribosome and is involved in the clearance of protein products generated both by NGD and NSD (Shao et al., 2013; Matsuda et al., 2014).

What role might Not4 play? Numerous studies have linked it to the protein QC on the ribosome. Firstly, the amount of Not4 on polysomes is increased in response to factors inducing cotranslational QC (Halter et al., 2014). Secondly, deletion of Not4 is accompanied by increased aggregation of polyubiquitinated and newly synthesized proteins, ribosome-associated chaperones, and ribosomal and proteasomal subunits (Halter et al., 2014). Thirdly, deletion of Not4 is accompanied by the accumulation of NAC in structures that are likely to be aggregated protein deposits (Panasenko et al., 2006). Forthly, the *not4*Δ mutant is extremely sensitive to agents that inhibit translation, or induce protein misfolding and proteotoxic stress (Halter et al., 2014). Fifthly, the products of translational arrest are stabilized in *not4*Δ cells and in a RING domain mutant of Not4 that has defective E3 ligase activity (Dimitrova et al., 2009). Finally, another RING domain mutant with impaired E3 ligase activity is sensitive to the proline analog, AZC that causes mistakes in proteins during translation (Halter et al., 2014). The last two observations support a requirement for the Not4 E3 ligase enzymatic activity in cotranslational QC. Surprisingly, however, ubiquitination of ribosome-associated translationally arrested polypeptides were increased in the absence of Not4, an observation that is inconsistent with a role in cotranslational ubiquitination (Duttler et al., 2013). The importance of Not4 for proteasome functional integrity may explain this last observation since proteasome inhibition or mutation also leads to the accumulation of polyubiquitinated protein products arising due to translational arrest (Wilson et al., 2007; Dimitrova et al., 2009; Bengtson and Joazeiro, 2010). This supports a role for Not4 in cotranslational protein QC via clearance of aberrant proteins via the proteasome. Mutants lacking both Not4 and Ltn1 have an aggravated slow growth phenotype (Halter et al., 2014) and increased accumulation of QC substrates, either full length or truncated (Bengtson and Joazeiro, 2010; Matsuda et al., 2014). The later observation points to synergistic roles for these E3 ligases with Ltn1 ubiquitinating the substrates and Not4 providing the functional proteasome.

## Not4 AND ITS REQUIREMENT FOR THE Ccr4-Not COMPLEX

The Ccr4-Not complex has deadenylation activity provided by the Ccr4/Caf1 module. Deletion of these subunits causes a strong accumulation of aberrant mRNAs (Basquin et al., 2012). It was suggested that Not4 might be necessary to activate the deadenylase module and, thus, activate mRNA degradation (Duttler et al., 2013). This may explain the accumulation of arrested proteins in cells lacking Not4. However, several studies suggest that Not4 does not activate the deadenylase of the Ccr4-Not complex [discussed in (Collart, 2013)]. The deadenylase module also does not regulate the Not4 E3 ligase (Panasenko and Collart, 2012; Halter et al., 2014). Thus, it is likely that Not4 acts in QC independently of Ccr4/Caf1 deadenylation function. In human and fly Not4 resides outside of the Ccr4-Not complex (Jeske et al., 2006; Lau et al., 2009) indicating that some functions of Not4 may not even require its association with the complex. However, in yeast we cannot exclude that the two modules of the Ccr4-Not complex might communicate to each other and be involved in cotranslational QC, participating both in mRNA and protein degradation.

## HYPOTHETICAL ROLE OF Not4 IN COTRANSLATIONAL QC

In our most recent study we did not observe an accumulation of truncated translationally arrested products produced from polybasic mRNA tracts when Not4 was deleted, probably because of decreased global translation (Halter et al., 2014). A decreased amount of full length QC substrates has been reported in the *not4Δ* background (Dimitrova et al., 2009; Bengtson and Joazeiro, 2010; Halter et al., 2014; Matsuda et al., 2014). These observations raise the possibility that Not4 may be important for preserving translation. In this scenario, Not4 might act either through the deadenylase module of the Ccr4-Not complex (Collart and Panasenko, 2012), or through the DEAD box RNA helicase Dhh1, with which the Ccr4-Not complex interacts (Maillet and Collart, 2002). Dhh1 represses translation in many different ways (Coller and Parker, 2005) and may also function as a “sensor” for slowed elongation (Sweet et al., 2012).

Another potential target for Not4 is the ribosome-associated chaperone NAC. NAC interacts very early with the nascent peptide emerging from the ribosome and participates in its cotranslational targeting (Zhang et al., 2012). The exposed peptides might be involved in the process of elongation pausing and thus, repression of protein synthesis, and chaperones such as Hsp70 and NAC might participate in this process (Shalgi et al., 2013). A role in translation repression was demonstrated for another ribosome-associated chaperone, RAC/Ssb (Chiabudini et al., 2012) and one could envisage that NAC might be also be involved in preserving translation. Ubiquitination of Rps7A by Not4 is important for cell viability. Ubiquitinated Rps7A was enriched in polysomal fractions (Panasenko and Collart, 2012). Thus, Rps7A ubiquitination by Not4 might be another requirement for normal translation. Finally, Not4 has an RRM and potentially can interact with mRNA and “sense” ribosome pausing and/or recruit the factors necessary for correct translation.

In conclusion, there is considerable data pointing to a role for Not4 in cotanslational QC. However, Not4 might act at multiple levels, although much remains to be clarified about its exact role in this complicated process.

## Conflict of Interest Statement

The author declares that the research was conducted in the absence of any commercial or financial relationships that could be construed as a potential conflict of interest.
